# Gandouling alleviates nerve injury through PI3K/Akt/FoxO1 and Sirt1/FoxO1 signaling pathway to inhibit autophagy in the rats model of Wilson's disease

**DOI:** 10.1002/brb3.3325

**Published:** 2023-11-27

**Authors:** Li Chen, Wangyang Xu, Yuting Zhang, Hao Chen, Yanquan Han

**Affiliations:** ^1^ Institute of Pharmaceutical Department The First Affiliated Hospital of Anhui University of Traditional Chinese Medicine Hefei P. R. China; ^2^ Institute of school of pharmacy Anhui University of Chinese Medicine Hefei P. R. China

**Keywords:** Gandouling, nerve injury, signaling pathway, Wilson disease

## Abstract

**Introduction:**

Previous studies have shown that Gandouling (GDL) may alleviate the nerve damage caused by Wilson's disease (WD) by inhibiting the autophagy of nerve cell mitochondria. However, its mechanisms are still unclear. Revealing the therapeutic mechanism of GDL is beneficial for its clinical application and provides theoretical support for the development of new formulations for treating WD.

**Method:**

This time we found that the oxidative stress level in the body of the copper‐overloaded WD rates increased, neurons in the hippocampus were damaged, and autophagy occurred. GDL reversed these situations and significantly improved the learning, memory, and spatial cognitive abilities of the high‐copper‐loaded WD rates. After GDL intervention, the expression of phosphatidylinositol‐3 kinase (PI3K), phosphorylated serine–threonine protein kinase (AKT), and phosphorylated forkhead box protein O1 (FoxO1) significantly increased, whereas FoxO1 in the nucleus decreased and phosphorylated FoxO1 in the cytoplasm also significantly raised. In addition, the expression of Sirt1 significantly declined, and Ac‐FoxO1 in the nucleus also significantly increased.

**Results:**

These data indicated that GDL may promote the phosphorylation of FoxO1 and promote its nucleation by activating the PI3K/AKT/FoxO1 signaling pathway and inhibit Ac‐FoxO1 hydrolysis in the nucleus through the Sirt1/FoxO1 signaling pathway to suppress the transcriptional activity of FoxO1.

**Conclusion:**

Furthermore, it inhibited the expression of autophagy genes Atg12 and Gabarapl1. In summary, our work provides new insights into the potential mechanisms of GDL repairing WD neuronal damage through autophagy pathways.

## Graphical abstract

In this work, we aimed to reveal the mechanisms of GDL alleviate nerve injury through PI3K/Akt/FoxO1 and Sirt1/FoxO1 signaling pathways to inhibit autophagy in the rat models of Wilson's disease. We prove that GDL may inhibit the malignant overexpression of Atg12 and Gabarapl1 through the PI3K/Akt/FoxO1 and Sirt1/FoxO1 pathways, thereby improving the neural function and improving nerve injury in the high‐copper‐induced WD rat model. These findings provide new insights into the mechanisms of GDL repairing neural function in WD patients and lay the foundation for our future research on the therapeutic mechanisms of GDL.

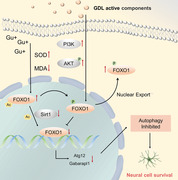

## 1 INTRODUCTION

Wilson's disease (WD) is a multisystem disease caused by the mutation of copper transport ATPase ATP7B, and the liver and nervous system are mainly involved (Scheiber et al., 2017). The Involvement of nervous system causes movement disorders, tremor, with symptoms of Huntington's chorea‐like rigidity and tremor, cerebellar dysfunction, hyperreflexia, seizures and cognitive impairment, and so on (Scheiber et al., 2017). If treatment measures are not taken in time, the patient's life will be endangered (Scheiber et al., 2017). At present, the main treatment methods are to block the intestinal absorption of copper (such as zinc‐containing preparations) and promote the elimination of copper (such as chelating agent), with significant effect (Lowette et al., 2010). However, they are accompanied by adverse reactions (ADRs), such as fever, gastrointestinal reaction, leukopenia, and systemic lupus erythematosus (Stremmel & Weiskirchen, 2021).

The d‐penicillamine (DPA) developed by John Walshe in 1956 is still recommended as a commonly used and effective drug for the treatment of WD (Członkowska et al., 2018). However, WD patients may experience ADRs, such as fever, gastrointestinal reactions, leukopenia, neurological deterioration, and systemic lupus erythematosus, during early (within the first 3 weeks after treatment introduction) or late (after 3 to several years of treatment) DPA copper therapy, which may ultimately result in 15%–30% of patients discontinuing treatment (Antos et al., 2021). Because about 20%–50% of patients with cerebral WD experience ADRs such as worsening brain symptoms during early treatment with DPA, European and American neurologists do not recommend DPA as the initial treatment for patients with cerebral WD (Yang, 2015).

Gandouling (GDL) is composed of six traditional Chinese medicines: *Rheum officinale* Baill. (Dahuang, DH), *Coptis chinensis* Franch. (Huanglian, HL), *Spatholobus suberectus* Dunn. (Jixueteng, JXT), *Curcuma longa* L. (Jianghuang, JH), *Salvia miltiorrhiza* Bge. (Danshen, DS), and *Curcuma phaeocaulis* Vai. (Ezhu, EZ), which was approved by Anhui Food and Drug Administration as the hospital preparation of the First Affiliated Hospital of Anhui University of Traditional Chinese Medicine in 2005 (Zhang et al., 2022). Early studies have shown the positive effects and mechanisms of GDL in the treatment of WD liver injury (Cheng et al., 2023; Liu et al., 2023; Zhang et al., 2021). Its protective effect on nerve injury has been proved in previous clinical, animal, and cell model studies (Dong et al., 2021; Zhang, 2019; Zhang et al., 2020). The mechanisms may be that GDL inhibits oxidative stress damage caused by high‐copper content by promoting the proliferation and regeneration of neural stem cells (Dong et al., 2021), inhibiting the autophagy of nerve cell mitochondria (Zhang et al., 2020), improving cerebrovascular injury, and reducing the secretion of vascular injury factors (Zhang, 2019). But the complex molecular mechanisms have not yet been fully clarified. So this study focuses on the mechanisms of GDL repairing neuronal damage. The homeostatic regulatory factors forkhead box protein O (FoxO) can trigger cell responses to environmental stimuli (such as oxidative stress response) to control redox status, genomic stability, and protein turnover, playing an important role in many cellular processes, such as apoptosis, autophagy, cell cycle inhibition, and inflammation (Calissi et al., 2021).

Studies have shown that the mammalian FoxO transcription factor is an important regulator of cell fate and function in the nervous system (Maiese, 2015). It can regulate the survival of nerve cells, stress response, lineage typing, and neuronal signal transduction and has become an important arbiter of the fate and function of nerve cells (Santo & Paik, 2018). There is a complex interaction between FoxO protein and its signal transduction pathway, which can significantly affect apoptosis and autophagy of programmed cell death pathway (Calissi et al., 2021). FoxO protein in mammals is composed of FoxO1, FoxO3, FoxO4, and FoxO6, of which FoxO1 is the most relevant to neuronal function. The widespread expression of FoxO1 may affect the survival of astrocytes, regulate ischemic brain injury, and the motor and memory pathways in the hippocampus striatum and subregions (Calissi et al., 2021). According to reports, AKT mediates FoxO1 phosphorylation through the phosphatidylinositol‐3 kinase (PI3K)/AKT pathway, preventing FoxO1 protein from entering the nucleus and promoting its nuclear output, thereby inhibiting FoxO1 transcription activity (Calissi et al., 2021). Acetylated FoxO1 can also promote FoxO1 phosphorylation and cytoplasmic localization. Sirt1 in the nucleus may activate the transcriptional regulatory activity of FoxO1 by the deacetylation of Ac‐FoxO1 (Calissi et al., 2021; Xu & Huang, 2014). The transcriptional activity of FoxO1 is mediated by the PI3K/AKT pathway and Sirt1/FoxO1 signaling pathway. The transcriptional activity of FoxO1 affects the expression level of autophagy‐related genes expression level of autophagy‐related genes Atg12 and Gabarapl1 (Xu & Huang, 2014).

Based on these evidences, we hypothesize that GDL might ameliorate PI3K/AKT/FoxO1 pathway and Sirt1/FoxO1 signaling pathway, interfere with the autophagy of nerve cells, and thus reduce the nerve damage caused by WD. To investigate this hypothesis, we investigated the possible molecular mechanisms of GDL in repairing WD nerve damage based on high‐copper‐loaded WD rats, which is a WD animal model that has been reported in many previous studies (Yu, 2020; Zhang et al., 2019).

## 2 MATERIALS AND METHODS

### 2.1 Animal models

Our animal trials were authorized by the Animal Ethics Committee of Anhui University of Traditional Chinese Medicine (ID: 2023‐015) on June 6, 2023 and were performed in the Animal Experiment Center of Anhui University of TCM following the use and care of Experimental Animals directions developed by the National Institutes of Health of the United States and the Prevention of Animal Abuse Act of China (1986). All male Wistar rats (4 months old, 180 ± 20 g, Jinan Pengyue Experimental Animal Breeding Co., Ltd., License No. SCXK 2019‐0003) adapted to the 12 h light–dark cycle environment of 22 ± 2°C, 55% ± 5% humidity for 7 days. GDL was produced by the First Affiliated Hospital of Anhui University of TCM (batch number 20210827). Anhydrous copper sulfate was ordered from Fuchen (Tianjin) Chemical Reagent Co., Ltd. (batch number 20211019).

**FIGURE 1:**
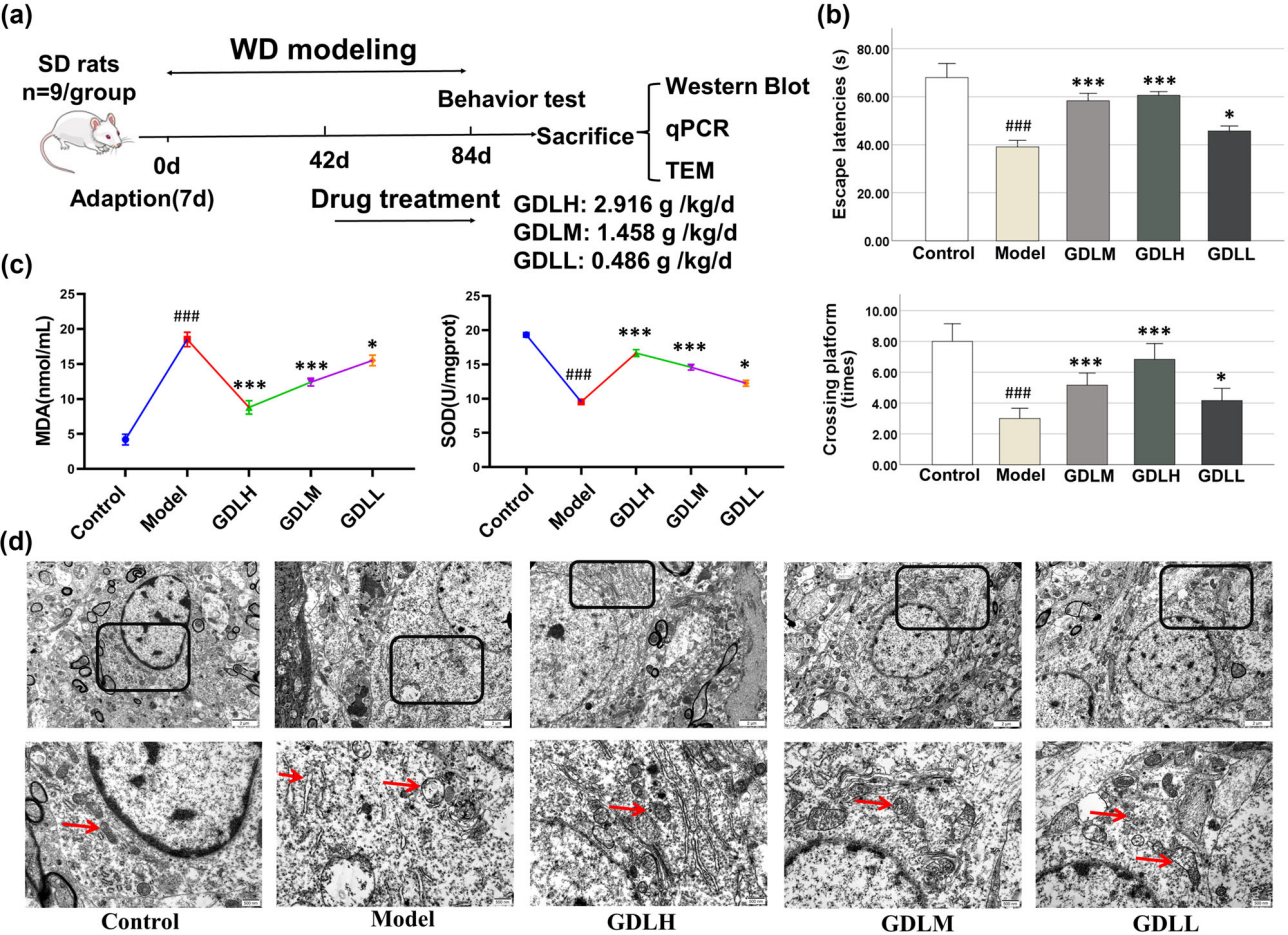
Gandouling (GDL) reduced oxidative stress, repaired nerve damage, and improved neural function in high‐copper‐loaded Wilson's disease (WD) rats: (a) schematic diagram of animal experiment (*n* = 9); (b) the behavioral evaluation results of Morris water maze test (*n* = 9); (c) the levels of malonic dialdehyde (MDA) and superoxide dismutase (SOD) in the hippocampus (*n* = 6); (d) mitochondrial ultrastructural changes in the hippocampal tissue CA1 region. All data expressed as the mean ± SD. ^###^ *p* < .001 versus control; *p < .05, ***p < .001 versus model.

This experimental procedure was performed as described in a previous report (Yu, 2020; Zhang et al., 2019). All the rats (*n* = 45) were grouped into the control normal group and model group. In the model group (*n* = 36), high‐copper diet induced neuronal damage. The normal group was fed with common feed. The model group was fed with 1 g/kg CuSO_4_ feed (Henan Huanyu Hekang Biotechnology Co., Ltd.) and 0.185% CuSO_4_ water every day. After 6 weeks, the 36 rats were divided into model group (*n* = 9), GDL high (GDLH)‐dose group (*n* = 9), GDL middle (GDLM)‐dose group (*n* = 9), and GDL low (GDLL)‐dose group (*n* = 9). They continued to be given a high‐copper diet with concurrent gavage for 6 weeks. During these 6 weeks, the GDLH, GDLM, and GDLL were given 2.916 g GDLH/kg/day, 1.458 g GDLM/kg/day, and 0.486 g GDLL/kg/day, respectively, whereas rats in the model and normal group were given 10 mL normal saline kg/day. The experimental design is depicted in Figure 1a.

### 2.2 Behavior testing

After 6 weeks of treatment, Morris water maze (MWM) test was carried out at Xin'an Medical Center of Anhui University of TCM to observe the effect of treatment on rats’ spatial learning and memory ability. The specific operation method of this experiment was referred to our published paper (Zhang et al., 2020). Before the start of the experiment, each rat was trained twice a day, with a rest of not less than 15 min between the two times, for a period of 4 days, and the time of the last arrival at the platform was collected. After that, we also conducted a space exploration experiment to collect the number of times that passed through the center of the pool in 120 s.

### 2.3 Transmission electron microscopy

The hippocampus was cut into 1 cm^3^ cubes, immersed in 2.5% glutaraldehyde at 4°C for 10 h, and immobilized in 1% osmium tetroxide for 2 h. The specimens were dehydrated in ethanol of various graded series and then soaked and embedded. Further, 70 nm ultrathin sections were cut and double‐stained with lead citrate and uranyl acetate. Used transmission electron microscope (JEM‐1400) to take pictures of organelle morphology and autophagy.

### 2.4 Biochemical testing

Enzyme‐linked immunosorbent assay was used to detect the changes of superoxide dismutase (SOD) and malonic dialdehyde (MDA) levels in rats. SOD and MDA test kits were obtained from the Nanjing Jiangcheng Bioengineering Research Institute (Nanjing, China). Used Elisa to detect the changes of rats’ CP (Recombinant Ceruloplasmin) level. CP kit was acquired from Wuhan Genmei Technology Co., Ltd. The kit's directions were followed for all trial procedures, and each trial was conducted three times in parallel.

### 2.5 Immunocytochemistry

The brain soaked in 10% formalin at 4°C overnight was removed for paraffin embedding and cut into 6 μm coronal section. The sections were incubated with rabbit acetylated FoxO1 (1:100; affinity) at 37°C for 60 min. Take out the sections and wash them with PBS‐T three times (Zsbia), and then incubate them with goat anti‐rabbit secondary antibody (Zsbia) at 37°C for 30 min. Wash the sections and add DAB dropwise (Zsbia) for color reaction. After dehydration, the sections were transparent with xylene (Shanghai Kaitong Chemical Co., Ltd). Images of the hippocampal CA1 region were collected using an optical microscope and quantified using Image Pro Plus 6.

### 2.6 Real‐time quantitative polymerase chain reaction (RT‐qPCR) analysis

Total RNA samples were extracted from the right hippocampus tissues using Trizol (Life Technologies,), and the concentration and purity of total RNA were determined by ultramicro spectrophotometry (Wuyi, Nanjing, China). The first strand cDNA is synthesized by the first strand cDNA synthesis kit according to the scheme of the manufacturer (TaKaRa). Real‐time polymerase chain reaction (RT‐PCR) system is used to detect quantitative RT‐PCR (Thermo Scientific). Based on the β‐reference expression of actin, the 2^−△△^ *^Ct^* method was used for the relative expression level of each gene. The primer sequences used for PCR were as follows: PI3K forward primer: 5′‐CGAAACAAAGCCGAGAACCT ‐3′; PI3K reverse primer: 5′‐GACGCAATGTTTGACTTCGC‐3′; AKT forward primer: 5′‐CAGGTTCACCCAGTGACAAC‐3′; AKT reverse primer: 5′‐CTCCTTCACCAGGATCACCT‐3′; Sirt1 forward primer: 5′‐TGACCTCCTCATTGTTATTGG‐3′; Sirt1 reverse primer: 5′‐ATTATGACATCGCAGTCTCCA‐3′; Gabarapl1 forward primer: 5′‐CAGCAACTCTCGTCACTTTG‐3′; Gabarapl1 reverse primer: 5′‐CAGCATTCTTTCCCCCTTTG‐3′; ATG12 forward primer: 5′‐CCCGTCTTCGGTTGCAGTTT‐3′; ATG12 reverse primer: 5′‐CGCTCCACAGCCCATTTCTT‐3′; beta‐actin forward primer: 5′‐CCCATCTATGAGGGTTACGC‐3′; beta‐actin reverse primer: 5′‐TTTAATGTCACGCACGATTTC‐3′.

### 2.7 Cytoplasmic and nuclear protein extraction

The Nuclear and Cytoplasmic Protein Extraction Kit (P0027 and P0028, Beyotime Institute of Biotechnology, Shanghai, China) was used to extract cytoplasmic and nuclear proteins from fresh hippocampal tissue. The extraction process strictly followed the instructions of the reagent kit. Extracts of cytoplasmic and nuclear proteins were used for subsequent experiments.

### 2.8 Western blot

The protein was extracted from hippocampus tissues using RIPA lysis buffer (1:100; Beyotime, Shanghai, China). Equal amounts of protein were separated using the SDS–polyacrylamide gel (SDS–PAGE) gel preparation kit (Solarbio). Electrophoresis was carried out on SDS–PAGE using 20 μg of total protein in each lane. After electrophoresis, the protein was transferred to a PVDF membrane. Then, the membrane was incubated with corresponding primary antibodies (Sirt1, 1:2000; Bioworld; PI3K, 1:1000; Bioss; AKT, 1:2000; Cell Signaling; p‐AKT, 1:1000; Cell Signaling; Gabarapl1, 1:1000; Bioss; FoxO1, 1:1000; Bioss; ATG12, 1:1000; Abcam; acetyl‐FoxO1, 1:2000; Thermo Fisher; p‐FoxO1, 1:1000; Bioss; GAPDH, 1:2000; Zsbio; LaminB1, 1:1000; Bioss) at 4°C overnight and the secondary antibody (1:20,000; Abcam) for 1 h, respectively. Eventually, the targeted antigens were detected by standard chemical luminescence methods. Band intensities were measured with Image software. During the experiments, we first detected the protein expression levels of Sirt1, PI3K, AKT, p‐AKT, FoxO1, ATG12, acetyl‐FoxO1, p‐FoxO1, and Gabarapl1 and then detected the protein expression levels of p‐FoxO1 in the cytoplasm and FoxO1 in the nucleus.

### 2.9 Statistical analysis

Quantitative data were expressed as the mean ± SD and analyzed with SPSS 22.0 software (International Business Machines Corporation). All data were analyzed using one‐way analysis of variance, followed by the least significant difference test or Tamhane's T2 test. A *p*‐value <.05 was considered statistically significant.

## 3 RESULTS

### 3.1 Effects of GDL on the behavior of copper‐loaded WD model rats

To observe the improvement of neurological deficits by GDL, we conducted MWM test on high‐copper‐loaded rats. The results are shown in Figure 1b. Compared with the control group, the latency of learning avoidance in the model group was significantly prolonged, and the frequency of crossing the platform was significantly decreased (*p* < .001). GDLM, GDLH, and GDLL groups significantly reduced escape latency and increased platform crossing frequency (GDLH: *p* < .001, GDLM: *p* < .001, GDLL: *p* < .05).

The result indicated that the learning, memory, and spatial cognitive abilities of high‐copper‐loaded rats significantly decreased, and the ability can be significantly improved after GDL intervention.

### 3.2 Effects of GDL on the levels of SOD and MDA in rats

As shown in Figure 1c compared with the control group, the SOD level in the model group decreased (GDLH: *p* < .001, GDLM: *p* < .001, GDLL: *p* < .05). Oxidative damage occurred in the model group. However, the level of MDA increased significantly (GDLH: *p* < .001, GDLM: *p* < .001, GDLL: *p* < .05). Compared with the model group, the SOD level increased (GDLH: *p* < .001, GDLM: *p* < .001, GDLL: *p* < .05), and MDA levels significantly decreased in the GDLM, GDLL, and GDLH groups (GDLH: *p* < .001, GDLM: *p* < .001, GDLL: *p* < .05). GDL repaired oxidative stress damage.

### 3.3 Effects of GDL on ultrastructure of hippocampal neurons in rats

Overdeposited free copper ions participate in oxidative stress reactions, induce excessive autophagy, and cause cell death and tissue damage (Samuele et al., 2005; Trejo‐Solís et al., 2012). The ultrastructural changes of neurons of hippocampus are shown in Figure 1d. Rich and healthy mitochondria, endoplasmic reticulum, and nuclei were observed in the control group. In the model group, organelles significantly decreased, mitochondria swelled or vacuolized, endoplasmic reticulum swelled and fractured, and mitochondrial phagosomes were visible. After drug intervention, the abnormalities of neuronal cell structure were significantly improved, in which GDLH and GDLM groups were significantly improved.

The results showed that GDL protected the hippocampal nerve damage of high‐copper‐loaded rats.

### 3.4 Effects of GDL on PI3K/Akt/FoxO1 signaling pathway

The activated PI3K in the PI3K/AKT signaling pathway catalyzes the production of the second messenger phosphatidylinositol‐3,4,5‐triphosphate (PIP3), which translocates the plasma membrane of AKT to trigger its phosphorylation. After that, PI3K/AKT activates the phosphorylation or complex formation of downstream molecules, including FoxO1 to promote the activation of downstream autophagy effectors (Calissi et al., 2021).

**FIGURE 2:**
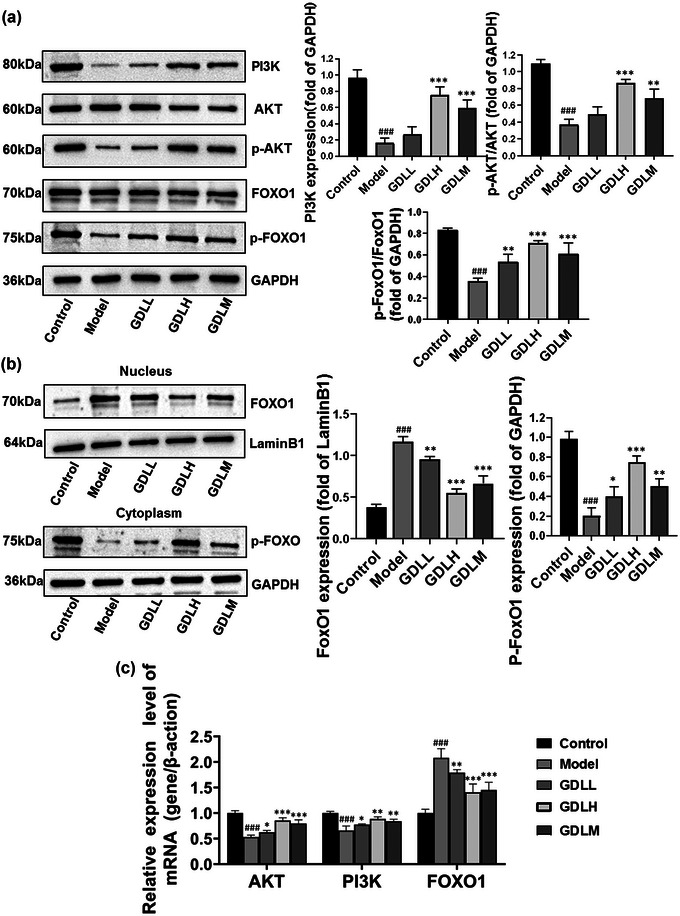
The effects of Gandouling (GDL) of phosphatidylinositol‐3 kinase/phosphorylated serine‐threonine protein kinase/forkhead box protein O1 (PI3K/AKT/FoxO1) pathway: (a) the protein levels of PI3K, p‐AKT, and p‐FoxO1 were detected by western blot in the hippocampus (*n* = 3); (b) the western blot was performed to detect the levels of p‐FoxO1 in cytoplasm and FoxO1 in nucleus (*n* = 3); (c) the relative mRNA expression levels of PI3K, AKT, and FoxO1 (*n* = 9). All data expressed as the mean ± SD. ^###^ *p* < .001 versus control; *p < .05, **p < .01, ***p < .001 versus Model. *Note*: The experiments of part (A) of this figure, Figure 3b, and Figure 4b were completed at the same time. The same internal reference GAPDH was used in the experiments, so the same GAPDH gel bands are used in part (A) of this figure, Figure 3b, and Figure 4b. In addition, part (A) of this figure and Figure 3b also use the same forkhead box protein O1 (FoxO1) gel bands, because both analyses of part (A) of this figure and Figure 3b use the total FoxO1 measured in the experiments.

In order to explore the effects of GDL on PI3K/Akt/FoxO1 signaling pathway in high‐copper‐loaded WD rats, western blotting was used to assess PI3K, AKT, p‐AKT, FoxO1, and p‐FoxO1 protein expressions (Figure 2a). The model group significantly decreased the expression levels of PI3K, p‐AKT, and p‐FoxO1 (*p* < .001), whereas GDL intervention could significantly reverse the levels of PI3K (GDLH: *p* < .001, GDLM: *p* < .001), p‐AKT (GDLH: *p* < .001, GDLM: *p* < .01), and p‐FoxO1 (GDLH: *p* < .001, GDLL: *p* < .01, GDLM: *p* < .001). As a transcriptional factor, FoxO1's nuclear localization is modulated by phosphorylation (Cheng et al., 2023). Therefore, FoxO1 nuclear translocation was studied by detecting the levels of p‐FoxO1 and FoxO1 in the cytoplasm and nucleus of hippocampal tissue, respectively. As exhibited in Figure 2b, the levels of p‐FoxO1 in the cytoplasmic protein extractions were strongly weakened in model group in contrast to the control (*p* < .001), and treatment with GDL increased the levels of p‐FoxO1 in the cytoplasm compared with model group (GDLH: *p* < .001, GDLL: *p* < .05, GDLM: *p* < .01). Correspondingly, the levels of FoxO1 in the nuclear fractions extracted from the hippocampus of the model group were significantly higher than those of the control group rats (*p* < .001). The uptrend of the FoxO1 was dramatically reversed by GDL (GDLH: *p* < .001, GDLL: *p* < .01, GDLM: *p* < .001). The gene expression levels of key molecules PI3K, AKT, and FoxO1in the hippocampus of rats were detected by RT‐qPCR. The results are shown in Figure 2c. Compared with the control group, the mRNA expression levels of AKT and PI3K were remarkably decreased (*p* < .001). In addition, GDL intervention could significantly reverse the mRNA levels of PI3K (GDLH: *p* < .01, GDLL: *p* < .05, GDLM: *p* < .01) and AKT (GDLH: *p* < .001, GDLL: *p* < .05, GDLM: *p* < .001). Compared with the control group, the mRNA expression levels of FoxO1 were remarkably increased (*p* < .001), whereas GDL intervention could significantly reverse the levels (GDLH: *p* < .001, GDLL: *p* < .01, GDLM: *p* < .001).

These data suggest that in high‐copper‐loaded rats, GDL may promote phosphorylation of FoxO1 through activating the PI3K/AKT pathway to promote nuclear excretion and thus inhibit FoxO1 transcriptional activity.

### 3.5 Effects of GDL on Sirt1/FoxO1 signaling pathway

**FIGURE 3:**
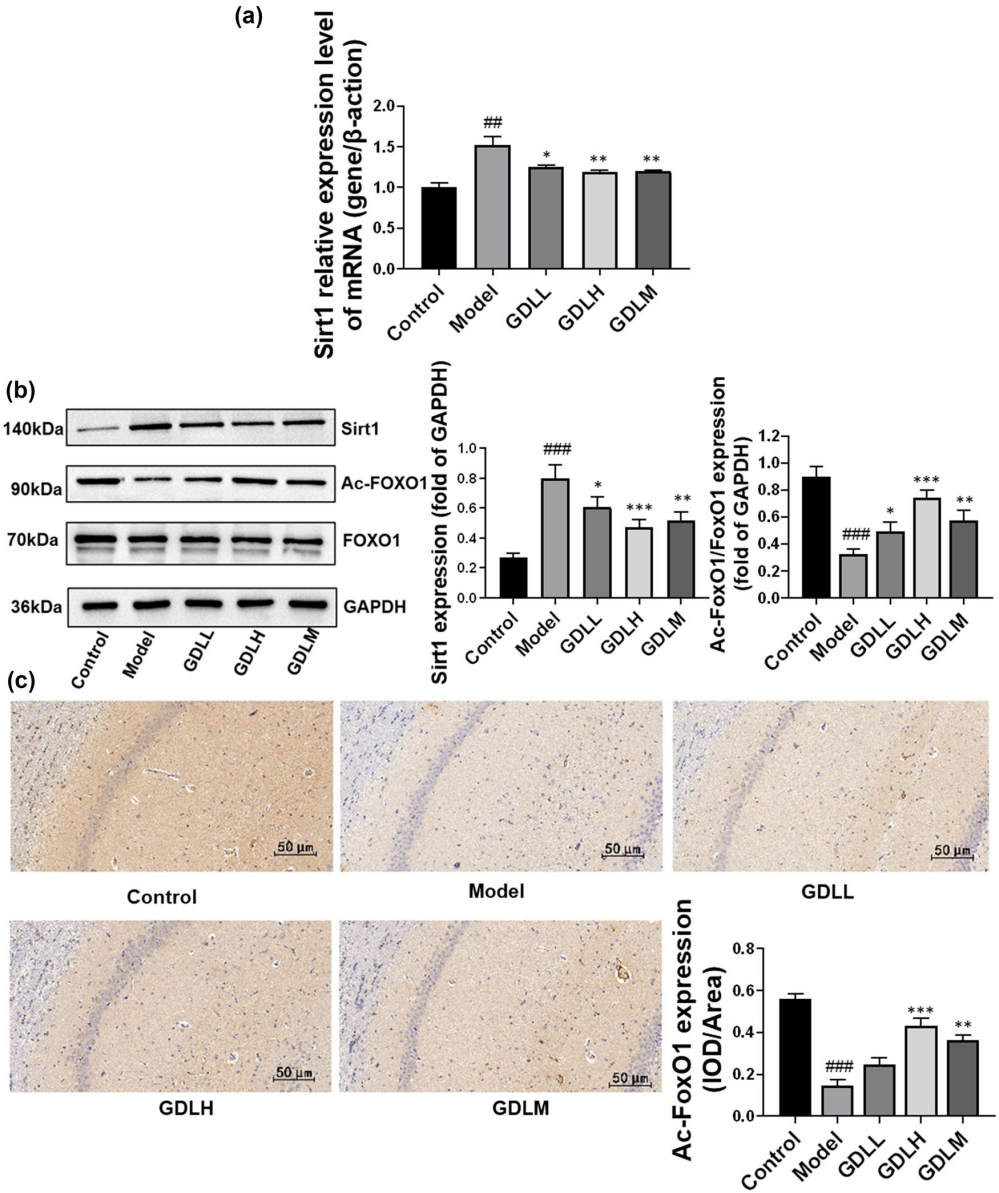
The effects of Gandouling (GDL) on Sirt1/forkhead box protein O1 (FoxO1) pathway: (a) the relative mRNA expression levels of Sirt1 (*n* = 9); (b) the Ac‐FoxO1 immunohistochemistry staining in the hippocampal CA1 subregion and its statistical analysis (scale bar 50 μm; *n* = 3 in each experiment); (c) the western blotting results of Ac‐FoxO1 and Sirt1 were quantified (*n* = 3). All data expressed as the mean ± SD. ^###^ *p* < .001 versus control; *p < .05, **p < .01, ***p < .001 versus model. *Note*: The experiments of Figure 2a, part (B) of this figure, and Figure 4b are completed at the same time. The same internal reference GAPDH was used in the experiments, so the same GAPDH gel bands are used in Figure 2a, part (B) of this figure, and Figure 4b. In addition, Figure 2a and part (B) of this figure also use the same FoxO1 gel bands, because both analyses of Figure 2a and part (B) of this figure use the total FoxO1 measured in the experiments.

Although during oxidative stress, FoxO1 is acetylated by CBP, p300, and so forth, which shields the transcription regulation activity of FoxO1, the Sirt1 in the nucleus can deacetylate Ac FoxO1 and activate the transcription regulation activity of FoxO1 (Xu & Huang, 2014). To explore the impact of GDL on the Sirt1/FoxO1 signaling pathway, the gene expression levels of Sirt1 were detected by RT‐qPCR. The results are shown in Figure 3a; compared with the control group, the mRNA expression levels of Sirt1 were remarkably increased (*p* < .01), whereas GDL intervention could significantly reverse the levels (GDLH: *p* < .01, GDLL: *p* < .05, GDLM: *p* < .01). Western blotting was used to assess Sirt1, FoxO1, and Ac‐FoxO1 protein expressions (Figure 3b). The model group significantly increased the expression levels of Sirt1 and Ac‐FoxO1 (*p* < .001), whereas GDL intervention could significantly reverse the levels of Sirt1 (GDLH: *p* < .001, GDLL: *p* < .05, GDLM: *p* < .01) and Ac‐FoxO1 (GDLH: *p* < .001, GDLL: *p* < .05, GDLM: *p* < .01). The Nissl training results are shown in Figure 3c. GDL significantly reversed the decrease in Ac‐FoxO1 induced by high copper in the hippocampal CA1 region (GDLH: *p* < .001, GDLM: *p* < .01).

These data indicate that in high‐copper load rats, GDL may inhibit the transcriptional activity of FoxO1 by inhibiting the deacetylation of Ac‐FoxO1 by Sirt1 in the nucleus.

### 3.6 Effects of GDL on mitophagy‐related protein expression

**FIGURE 4:**
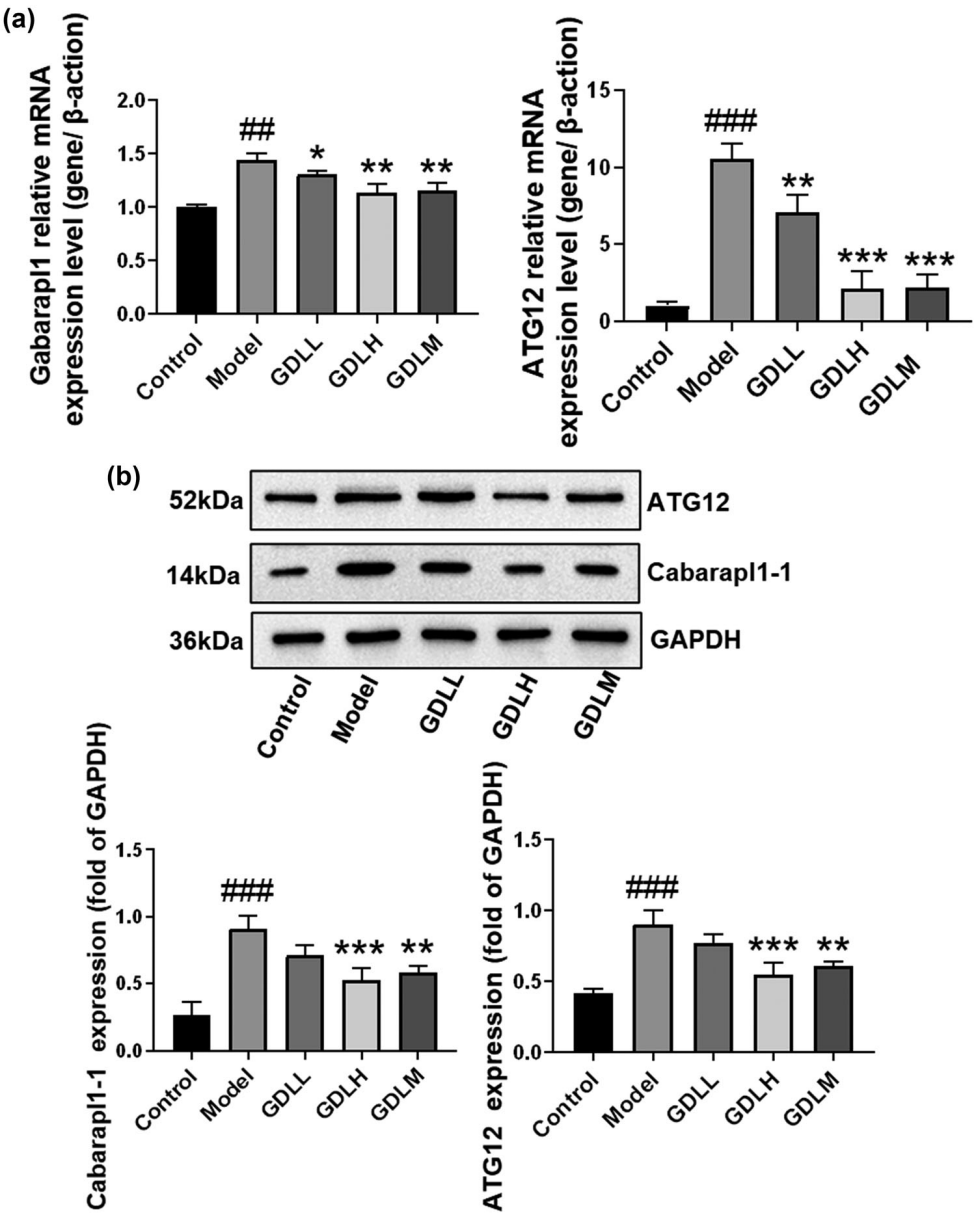
The effects of Gandouling (GDL) on Atg12 and Gabarapl1 autophagy‐related in high‐copper‐loaded Wilson's disease (WD) rats: (a) the relative mRNA expression levels of Atg12 and Gabarapl1; (b) the western blotting results of Atg12 and Gabarapl1 (*n* = 3). All data were presented as means ± SD. ^###^ *p* < .001 versus control; *p < .05, **p < .01, ***p < .001 versus Model. *Note*: The experiments of Figure 2a, Figure 3b, and part (B) of this figure are completed at the same time. The same internal reference GAPDH was used in the experiments, so the same GAPDH gel bands are used in Figure 2a, Figure 3b, and part (B) of this figure.

The transcription factor FoxO1 can affect the degree of autophagy by regulating the expression levels of autophagy‐related genes Atg12 and Gabarapl1 (Xu & Huang, 2014). The above research results indicate that GDL can inhibit the transcription activity of FoxO1 through PI3K/AKT pathway and Sirt1/FoxO1 signaling pathway. To investigate the indirect intervention of GDL on autophagy‐related gene expression, RT‐qPCR was used to detect the gene expression levels of Atg12 and Gabarapl1 (Figure 4a); compared with the control group, the mRNA expression levels of Atg12 and Gabarapl1 were remarkably increased (*p* < .001, *p* < .01, respectively), whereas GDL intervention could significantly reverse the levels of Atg12 (GDLH: *p* < .001, GDLL: *p* < .01, GDLM: *p* < .001) and Gabarapl1 (GDLH: *p* < .01, GDLL: *p* < .05, GDLM: *p* < .01). Western blotting was used to assess Atg12 and Gabarapl1 protein expression (Figure 4b). Compared with the control group, the expression levels of Atg12 and Gabarapl1 were remarkably increased (*p* < .001), whereas GDL intervention could significantly reverse the levels of Atg12 (GDLH: *p* < .001, GDLM: *p* < .001) and Gabarapl1 (GDLH: *p* < .001, GDLM: *p* < .01). Altogether, these results suggest that GDL could inhibit FoxO1 transactivation via the activation of PI3K/AKT signaling and Sirt1/FoxO1 signaling pathway to inhibit the expression of Atg12 and Gabarapl1 in high‐copper‐loaded rats.

## 4 DISCUSSION

Neurological impairment is the most important clinical manifestation of WD disease, accounting for over 50% of the total cases (Dusek et al., 2019; Litwin et al., 2019). Anti‐copper treatment for WD requires treatment monitoring of patients to prevent serious adverse events, especially early neurological deterioration (Antos et al., 2023). The monitoring and evaluation of neurological symptoms using the Unified Wilson's Disease Rating Scale or the Global Assessment Scale for WD, as well as quantitative monitoring of blood biomarkers for central nervous system injury in WD (such as neurofilament proteins, glial fiber acid protein, tau proteins, and ubiquitin carboxyl terminal hydrolase L1), are effective methods for preventing early neurological deterioration (Ziemssen et al., 2022, 2023). But if there is a drug that can improve the neurological symptoms of cerebral WD and prevent the deterioration of neurological symptoms, it will effectively increase the cure rate of WD patients, especially those with cerebral WD.

Numerous clinical observations have shown that GDL has such therapeutic advantages. Zhang et al. evaluated the efficacy and safety of GDL with sodium dimercaptosulfate (DMP) and GDL with low‐dose DPA in patients with WD in the nervous system in 2018 (Zhang, Xie, et al., 2018; Zhang, Li, et al., 2018). The results showed that the effective rate of significantly improving neurological symptoms (both reaching over 90%) and the incidence of neurological deterioration were significantly lower than those using sodium DMP or DPA alone. Bao TT's research found that the effective rate of GDL in treating patients with cognitive impairment in WD is equivalent to that of Olaxitam (over 80%), significantly reducing the vascular dementia assessment scale log score and activities of daily life index, indicating that GDL can significantly improve cognitive impairment in WD and enhance daily living ability (Bao, 2015). Wang's (2021) research found that GDL can significantly increase the mini–mental state examination and monthly cognitive assessment scale scores in patients with mild cognitive impairment in WD, which may be related to a decrease in the serum levels of brain‐derived neurotrophic factor and vascular endothelial growth factor in patients. Those indicate that GDL significantly improves the safety and effectiveness of anti‐copper therapy in WD patients.

Studies have shown that the effect of GDL on the recovery of neurological function in WD patients may be related to the inhibition of excessive autophagy of neurons (Zhang et al., 2020). This study found that GDL improved the learning, memory, and spatial cognitive abilities of high‐copper‐loaded WD rats. Its potential mechanism may be related to the PI3K/Akt/FoxO1 and Sirt1/FoxO1 signaling pathways. According to reports, free copper may cross the blood–brain barrier and induce oxidative stress in WD with neurological manifestations (Choi & Zheng, 2009). WD patients with neurological symptoms have significantly higher levels of oxidative stress and lower levels of antioxidant stress compared to liver‐type and asymptomatic WD patients (Bruha et al., 2012). In addition, when neurological symptoms worsen in WD patients, oxidative stress activity increases and antioxidant stress levels decrease (Kalita et al., 2014, 2015). The above indicates a high correlation between cerebral WD and oxidative stress. In the oxidative stress process, transcription factors FoxO may be activated to coordinate responses to environmental changes and trigger autophagy (Obsil & Obsilova, 2008), so we speculate that excessive autophagy induced by FoxO may play a role in WD neurological dysfunction. In our study, the WD model rats induced by a high‐copper diet showed a significant decreased in SOD levels and a significant increase in MDA levels in the hippocampus. However, the administration of GDL significantly improved the neural function of model rats and significantly reduced oxidative stress levels while downregulating the expression of FoxO1. These data indicated that GDL can repair neural function, and FoxO1 may be involved in this process.

The FoxO family, an important arbiter of neuronal fate and function, has been found to regulate neuronal survival, stress response, lineage shaping, and neuronal signaling (Maiese, 2015). In the FoxO family, FoxO1 is most correlated with neuronal function (Santo & Paik, 2018). FoxO1 activity is regulated at the transcriptional level, and its transcriptional activity is regulated by posttranslational modifications (PTMs) (Calissi et al., 2021). Phosphorylation is the main predominantly through PTM that regulates FoxO1 activity (Calissi et al., 2021). The phosphorylation of FoxO1 by PI3K/AKT can prevent FoxO1 protein from entering the nucleus, promote its nuclear output, and thus block DNA binding, thereby inhibiting the expression of target genes (Calissi et al., 2021). In this study, GDL treatment significantly increased the expression of PI3K and p‐AKT and then promoted the phosphorylation of FoxO1, which facilitates FoxO1 nuclear expulsion, as shown in Figure 2. Therefore, GDL may inhibit the activity of FoxO in high‐copper‐loaded WD rats by activating the PI3K/AKT/FoxO1 pathway, especially GDLH.

During oxidative stress, FoxO protein is acetylated and deacetylated by Sirt1 (Motta et al., 2004). Previous studies have suggested that the acetylation and deacetylation of FoxO do not act as on/off regulators of FoxO activity, similar to phosphorylation (Brown & Webb, 2018). But Ac‐FoxO in the nucleus may be deacetylated by Sirt1, thereby activating the transcriptional regulatory activity of FoxO to induce autophagy (Glauser & Schlegel, 2007). As shown in Figure 3, the transcription and expression levels of Sirt1 significantly increased, whereas the expression level of Ac‐FoxO1 decreased in high‐copper‐loaded WD rats. Combined with the increased transcription level of FoxO1 in Figure 2c, we speculated that the transcription activity of FoxO1 in the model group is activated through the Sirt1/FoxO1 signaling pathway, which may induce autophagy. After GDL treatment, these trends were reversed, especially in GDLH. These results indicated that GDL may inhibit the transcriptional activity of FoxO1 through the Sirt1/FoxO1 signaling pathway. In addition, the increased Ac‐FoxO1 may help FoxO1 to be phosphorylated by Akt (Nasrin et al., 2009), resulting in synergistic effects.

The above research indicated that GDL may reduce the transcription level of FoxO1 and limit its transcription activity. It is reported that the transcriptional activity of FoxO1 regulates the expression level of autophagy‐related genes Atg12 and Gabarapl1 (Calissi et al., 2021). In Figure 4, the transcription and expression levels of autophagy‐related genes decreased after GDL treatment. This indicated that GDL has decreased autophagy levels, which may be related to PI3K/Akt/FoxO1 and Sirt1/FoxO1 signaling pathways.

## 5 CONCLUSIONS

**FIGURE 5:**
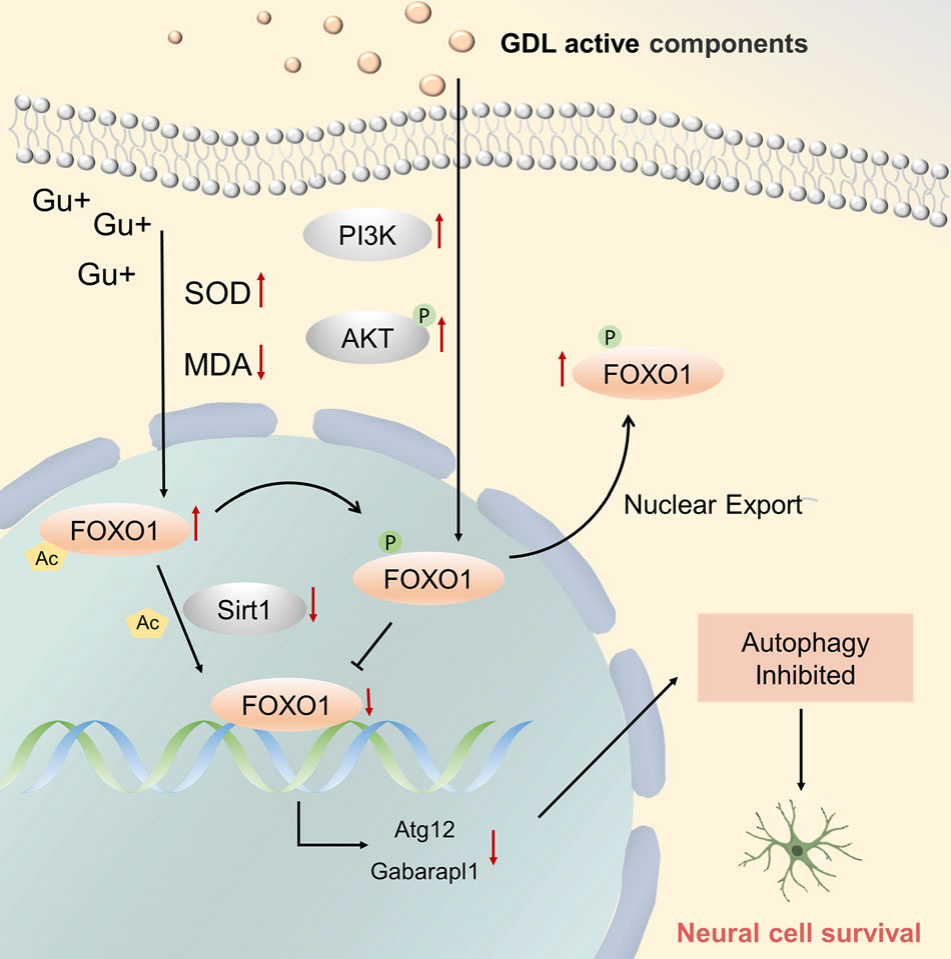
Schematic diagram of the mechanism of Gandouling (GDL) repairing Wilson's disease (WD) neural function by inhibiting excessive autophagy.

The proposed pathway is shown in Figure 5. We prove that GDL may inhibit the malignant overexpression of Atg12 and Gabarapl1 through the PI3K/Akt/FoxO1 and Sirt1/FoxO1 pathways, thereby improving the neural function and improving nerve injury in the high‐copper‐induced WD rat model. These findings provide new insights into the mechanisms of GDL repairing neural function in WD patients and lay the foundation for our future research on the therapeutic mechanisms of GDL.

## AUTHOR CONTRIBUTIONS

All authors contributed to the study conception. Li Chen, Hao Chen, and Yanquan Han conceived and designed the experiment. Li Chen performed the experiments. Li Chen, Wangyang Xu, Yuting Zhang, and Hao Chen analyzed the data. Li Chen and Yanquan Han wrote the paper. All of the authors reviewed and approved the submitted version of the paper.

## CONFLICT OF INTEREST STATEMENT

The authors declare no possible conflicts of interest or financial situation in this investigation.

### PEER REVIEW

The peer review history for this article is available at https://publons.com/publon/10.1002/brb3.3325.

## Data Availability

All data involved in this study are included in this paper.
